# Detection of class 1 integron-associated gene cassettes and tetracycline resistance genes in *Escherichia coli* isolated from ready to eat vegetables

**DOI:** 10.1016/j.amsu.2020.04.044

**Published:** 2020-05-21

**Authors:** Saad A. Mohamed, Tri Ardiyati, Muhaimin Rifa’i, Widodo

**Affiliations:** a Biology Department, Faculty of Sciences, Bani Walid University, Libya; b Biology Department, Faculty of Mathematics and Natural Sciences, Brawijaya University, Indonesia

**Keywords:** Tetracycline-resistant *E. coli*, Multi-drug resistance, Ready to eat vegetables, Class 1 integron, dfrA7

## Abstract

**Background:**

Ready to eat (RTE) vegetables are easily accessible healthy foods that are commonly consumed globally, including in Indonesia. However, these RTE vegetables contain potential contamination from pathogens and multi-drug resistant bacteria. Therefore, in the present study, we examined the presence of tetracycline-resistant *E. coli* (TRE) isolates from RTE vegetables.

**Methods:**

Susceptibility to antimicrobial agents was determined using the Kirby-Bauer disc diffusion method. Characterisation of antibiotic resistant genes was performed using PCR and sequencing of tetracycline resistant gene, integron and gene cassette from the TRE isolates.

**Results:**

The isolates collected in this study were resistant not only to tetracycline, but also to streptomycin. Some isolates also displayed resistance to kanamycin (77.8%), chloramphenicol (11.1%), and ciprofloxacin (5.6%). All of the isolates contained integrons (*intI1*) and the *tetA* gene; *tetB* was not detected in our study. Further analysis showed that some isolates (38.8%) contained the *dfr*A*7* gene cassette, which encodes dihydrofolate reductase, which is responsible for resistance to trimethoprim. Of all the isolates that presented integrons, 11 isolates (61.1%) did not carry gene cassettes. These empty integrons have the potential to convert themselves rapidly into multigraviton strains.

**Conclusions:**

TRE isolates contain the *tetA* gene and integron 1. Only 38.8% of the isolates that have been identified contain the *dfrA7* gene cassette, which is responsible for trimethoprim antibiotic resistance. Further identification of genes conferring resistance to other antibiotics is necessary to better characterise antibiotic resistance.

## Highlights

• This study was obtained Tetracycline Resistant *E. coli* (TRE) isolate.
• The isolate was also resistant to streptomycin.
• The isolate contains integrons (*intI1*) and the *tetA* gene.

## 1 Introduction

Ready to eat (RTE) vegetables are commonly consumed in the modern world as they are low in calories, high in fibre and provide intake of vitamins, minerals, and other phytochemicals [1]. Fresh food is regularly colonised by a wide assortment of microscopic pathogenic organisms [2]. These microscopic organisms have the potential to lead to public health problems, as many are pathogenic to humans, such as *E. coli*. These pathogens are bind to plant leaves and can be internalised in the leaves or the endophytic root system [3]. RTE vegetables are commonly consumed in Indonesia. In order to determine the prevalence of antibiotic resistant *E. coli* in RTE vegetables, we collected and characterised isolates collected from RTE vegetables.

Antimicrobial resistance in humans and other living organisms is a global public health concern. Broad utilisation of antimicrobials leads to the increase and spread of resistant microorganisms, especially multidrug-resistant microscopic organisms. Broad utilisation of antimicrobials also leads to resistance to newly developed compounds, such as, the tetracycline class antimicrobials [4,5]. Tetracycline is classified in a group of an expansive range of antibiotics that are used for cultivating domesticated animals and aquaculture. Excessive use of tetracycline has led to increased tetracycline-resistant microbes.

The primary mechanisms of tetracycline resistance in microorganisms is a functioning efflux system, ribosomal protection, and inactivation of enzymes [6]. The most widely recognised mechanism of tetracycline resistance in gram-negative bacteria is the energy-dependent efflux pump system, which is encoded by *the tetA, tetB, tetC*, *tetD* and *tetG* genes. Studies suggest that antibiotic resistant genes can be transferred from microflora nourishment to pathogenic bacteria leading to antibiotic resistance in these bacteria [7]. The exchange of resistance genes situated on versatile DNA components, for example integrons, is a common mechanism for the development of antibiotic resistance [8].

Multidrug resistance (MDR) in intestinal microscopic organisms, such as *E. coli* is associated with integrons [9], which are a main factor involved in the spread of multidrug resistance [10]. Integrons were characterised by Hall and Collis as DNA components that work as quality catch and articulation frameworks [11,12]. Integrons consist of three vital segments, including an integrase gene (*intI*), which defines a site-specific recombinase enzyme, an attI site, which is detected by the integrase and acts as an acceptor for quality cassettes, and a promotor region (PC) [9]. There are three kinds of integrons, each with various *intI* genes, (*intI1, intI 2* and *intI 3*). Class 1 integrons have been documented in various gram-negative bacteria, including acinetobacter, Salmonella, Alcaligenes, Campylobacter, Vibrio, Aeromonas, Proteus, Burkholderia, Enterobacter, Citrobacter, Mycobacterium, Pseudomonas, Serratia, Klebsiella, Shigella and Escherichia [13]. Despite the fact that integrons are not portable, they can be exchanged between microorganisms via transposons or plasmids.

## 2 Materials and methods

### 2.1 Isolation and identification of tetracycline-resistant *E. coli* (TRE)

Eighteen TRE isolates and several identified in our previous study [14] were used for the current. Six isolates were from lettuce, eight isolates were from lemon basil, three isolates were from yard long beans, and one isolate was from cabbage. All 18 isolates of TRE were subjected to antibiotic susceptibility testing in which the Kirby-Bauer disc diffusion method was used. Antibiotic commonly used such as chloramphenicol (30 μg), gentamycin (10 μg), streptomycin (10 μg), tetracycline (30 μg), ciprofloxacin (5 μg), and kanamycin (30 μg) (Oxoid, England) were tested. The susceptibility test was administered using Mueller-Hinton agar and McFarland 0.5 from overnight cultures, followed by an incubation at 36 °C for 18–20 h. After incubation, inhibition zone diameters were determined with a millimetric ruler. The inhibition zone diameters were interpreted according CLSI guidelines [15].

### 2.2 Detection of tetracycline resistance genes and class 1 integrons

Extraction of DNA was performed according to the manufacturer's instructions (Presto Mini gDNA Bacteria Kit, Geneaid Biotech, Ltd., Taiwan). The DNA quality and quantity were evaluated and measured by an absorbance spectroscopy at 260 and 280 nm and then the extracted DNA was stored at −20 °C.

TRE isolates were examined for the presence of *tet* resistance genes (*tetA, tetB*) by PCR using primer sequences (Table 1). PCR was performed on the 8800 thermal cycler (Agilent, USA). Each reaction contained a total volume of 25 μl, containing 12.5 μl of 2X PCR for KOD FX neo (Toyobo, Japan), 0.5 μl of each primer (Integrated DNA Technologies (IDT) Pte. Ltd. Singapore), 1 μl of DNA template, 5 μl of 2 mM dNTPs, 0.5 μl of KOD FX Neo (1.0U/μl), and 5 μl of nuclease free water. The cycling conditions for the PCR reaction consisted of an initial denaturation step (94 °C, 2 min), followed by 35 cycles of denaturation (98 °C, 15 s), annealing of primers (59 °C for 30 s), and an extension step (68 °C, 45 s). The amplified PCR products were analysed by electrophoresis on 1.5% w/v agarose gels in TBE buffer, pH 8.3 (1st Base, Malaysia) and were stained with 0.5 μg/mL of ethidium bromide and visualised using a UV transilluminator. The class 1 integron was detected according to previous protocol [16].

**Table 1:** Primers used in this study.

Primer	Target region	Sequence	Product size (bp)	Reference
hep58	Variable region	5′-TCATGGCTTGTTATGACTGT	Variable	[16]
hep59		5′-GTAGGGCTTATTATGCACGC		
IntI1F	Class 1 integron	5′-GGGTCAAGGATCTGGATTTCG	483	[30]
IntI1R		5′ACATGCGTGTAAATCATCGTCG		
*tet*A-FW	*tet*A	5′-GCTACATCCTGCTTGCCTTC	210	[17]
*tet*A-RV		5′-CATAGATCGCCGTGAAGAGG		
*tet*B-FW	*tet*B	5′-TTGGTTAGGGGCAAGTTTTG	659	[17]
*tet*B- RV		5′-GTAATGGGCCAATAACACCG		

### 2.3 PCR and sequencing for detection of gene cassettes

The primers used to amplify the class 1 integron cassette region were: hep58 and hep59 (Table 1). PCR ampliﬁcations were carried out in 25 μl reaction mixtures containing 10.5 μl of dd H_2_O, 12.5 μl of DreamTaq Green PCR master mix, 0.5 of each primer, and 1 μl of DNA template. DNA amplification was performed in accordance with the following steps of PCR reaction: initial denaturation at 95 °C for 3 min, followed by 35 cycles of denaturation at 94 °C for 15 s, primer annealing at 54.7 °C for 30 s, and extension at 72 °C for 45 s.

The PCR product was purified using the DNA Clean & Concentrator kit (Zymo Research, USA) and cycle sequencing using forward and reverse primers (IDT Pte. Ltd. Singapore) was performed using the BigDye Terminator v3.1 Cycle Sequencing Kit. (Applied Biosystems, USA) in an automated DNA sequencer (ABI 3730xl, Applied Biosystems). The amplicons were purified and sequenced at the 1st Base, Malaysia.

Sequences were edited with DNAMAN Version 9 (Lynnon BioSoft, USA) and analysed using the BLAST programme of the NCBI database http://www.ncbi.nlm.gov/blast.

## 3 Results

### 3.1 Susceptibility testing

Susceptibility testing of the 18 TRE isolates showed that all isolates were resistant to both tetracycline and streptomycin (100%). 14 isolates (77.8%) were also resistant to Kanamycin. 2 isolates were resistant to chloramphenicol (11.1%) and 1 isolate was resistant to ciprofloxacin (5.6%).No resistance to gentamycin was observed. The isolates showed intermediate resistance to gentamycin (77.7%), ciprofloxacin (22.2%), kanamycin (11.1%), and chloramphenicol (5.5%). The percentages of samples that were susceptible to the antibiotics are as follows: chloramphenicol (83.3%), ciprofloxacin (72.2%), and gentamycin (22.2%) (Table 2).

**Table 2:** Antimicrobial Susceptibility Test of 18 TRE isolates.

NO	Isolate ID	Source	TET	CIP	GEN	STR	KM	CHL
1	L1.1	Lettuce	R	S	S	R	I	S
2	L1.2	Lettuce	R	S	I	R	R	S
3	L1.3	Lettuce	R	I	I	R	R	S
4	L3.3	Lettuce	R	S	I	R	R	R
5	L3.2	Lettuce	R	I	S	R	R	R
6	L3.1	Lettuce	R	S	I	R	R	S
7	B1.2	Basil	R	S	I	R	R	S
8	B1.1	Basil	R	S	I	R	R	I
9	B2.1	Basil	R	I	S	R	S	S
10	B2.2	Basil	R	S	I	R	S	S
11	B2.3	Basil	R	S	I	R	R	S
12	B3.1	Basil	R	S	I	R	R	S
13	B3.3	Basil	R	R	I	R	R	S
14	B5.1	Basil	R	S	I	R	R	S
15	Y1.1	Yard long bean	R	S	I	R	R	S
16	Y1.3	Yard long bean	R	S	I	R	I	S
17	Y3.3	Yard long bean	R	I	I	R	R	S
18	C3.1	Cabbage	R	S	S	R	R	S

Note: R is resistant; S is sensitive; I is intermediate

### 3.2 Detection of class 1 integron and tetracycline resistance genes

**Fig. 1:**
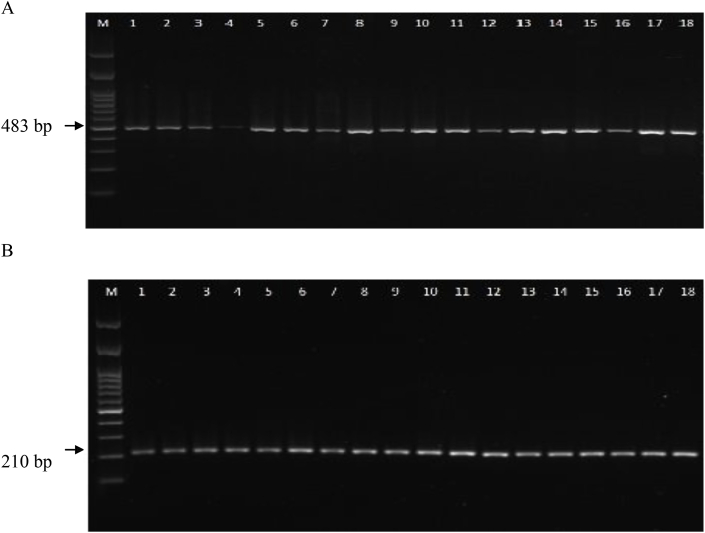
Characterisation of Antibiotic Resistance Gene Cassettes in Class 1 Integrons. All TRE isolates contain class I integrons, shown by the PCR product bands at 483 bp (A); and contain the *tetA* gene shown by the PCR product at 210 bp (B). M is DNA marker; number of isolate was marked by number 1-18.

**Fig. 2:**
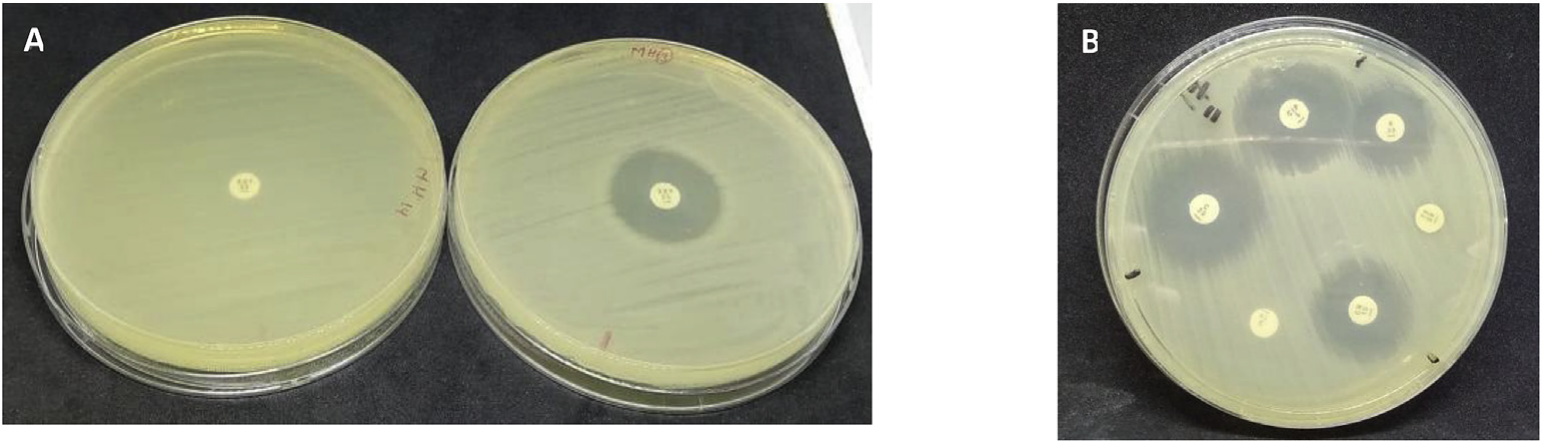
Antibiotic susceptibility test. TRE showed resistance (left side) and were sensitive to trimethoprim*/*sulfamethoxazole (right side) (A), other antibiotics listed in Table 1 (B).

The results of the study showed that all 18 (100%) of the isolates contain class 1 integrons, as indicated by the PCR product size of 483 bp (Fig. 1). In addition, all isolates were resistant to tetracycline, streptomycin, and kanamycin (Table 2). We next further characterised the tetracycline resistant genes and found that all TRE isolates contain the *tetA* gene, rather than the *tetB* gene, shown as the from PCR product with the size of 210 bp, and confirmed by sequencing (Fig. 2, Table 3).

**Table 3:** Identification of the *tet* gene, integron and gene cassette of TRE isolates.

No	Source	*tet A/tet B Gene*	Integron Type	Gene Cassette
1	Lettuce	*tetA* (100%)	*intI1* (100%)	*dfrA7* (66.66%)
2	Basil	*tetA* (100%)	*intI1* (100%)	*dfrA7* (12.50%)
3	Long bean	*tetA* (100%)	*intI1* (100%)	*dfrA7* (33.33%)
4	Cabbage	*tetA* (100%)	*intI1* (100%)	*dfrA7* (100%)

We used hep58 and hep59 primers to amplify the gene cassette on the integron 1 (*intI1*)-positive isolates using PCR. 7 of 18 (38.8%) *intI1*-positive isolates contained amplicons of 800 bp. These amplicons were then sequences via sanger sequencing and all of the amplicons matched the *dfrA7* cassette gene (Table 3). Sequence alignment showed 100% identity with the *E. coli* strain MA26d class 1 integron (accession no. MF465028). The *dfrA7* cassette confers resistance to trimethoprim. We then assessed whether the isolates were also resistant trimethoprim and found that these *dfrA7*-positive isolates were resistant to trimethoprim.

## 4 Discussion

In this study, all of the TRE isolates were resistant to at least three antibiotics. This is similar to the rate of MDR reported in *E. coli* isolates collected by Thorsteinsdottir et al., 2010 [18].

TRE isolates from this study were not only resistant to tetracycline, but were also all resistant to streptomycin, and many were also resistant to Kanamycin (77.8%), chloramphenicol (11.1%), and ciprofloxacin (5.6%). The study suggests that TRE isolates displayed MDR, which corresponds with previous reports that suggest that streptomycin resistance was present in 100% of MDR cases [19]. The percentages of chloramphenicol and ciprofloxacin resistant isolates from this study are consistent with previous findings [20].

As we found that the isolates were resistant to tetracycline, we then aimed to investigate which tetracycline resistant gene the isolates contained, using PCR. All of the isolates contained the *tetA* gene and *tetB* was not detected in our study. This observation is consistent with previous reports that found that 96% of samples isolated from vegetables harboured *tetA* [21]. However, these results contrast with previous reports suggest that TRE isolates contain a higher frequency of *tetB* [22]. According to Sengeløv et al., 2003 [23], the rapid diffusion of tetracycline resistance genes to bacteria is due to the localisation of the *tetA* gene on plasmids, transposons and integrons. In addition, the *tetA* and *tetB* genes are present in soil and water for long periods of time [24].

Integrons are mobile gene elements that contain two conserved segments and a central variable gene cassette that commonly encodes for antibiotic resistance. Four integron types have been defined, but the majority of integrons found in clinical isolates belong to class 1 [25]. In the present study, all TRE isolates were positive for class 1 integrons, which is higher than the rate reported for *E. coli* isolated from cooked meat in China (14.7%) [26]. All intI1-positive isolates showed resistance to three or more classes of antimicrobials. We then assessed the gene cassette that was responsible for the resistance to the antibiotic. We found that the dfrA7 cassette, which encodes dihydrofolate reductase, was identified in 7 (38.8%) isolates of the 18 TRE isolates that contained integron class 1. These results are similar to what has been reported previously from other *E. coli* isolates [27].

Of all the isolates that presented integrons, 11 isolates (61.1%) did not carry gene cassettes; these are called empty integrons. Empty integrons have been observed in other studies [28], suggesting that these bacteria can have the potential to rapidly convert themselves into multi-resistant strains. Other studies have proposed that these ‘‘empty’’ integrons represent ancestral elements that have not yet acquired gene cassettes, that are inserted between the conserved segments of the integrons [29].

## 5 Conclusions

In conclusion, all of TRE isolates contained the *tetA* gene and integron 1, and were resistant to tetracycline and streptomycin. However, only 38.8% of the isolates that were identified contain the *dfrA7* gene cassette which is responsible for resistance to trimethoprim. Further identification of genes responsible for resistance to other antibiotics is necessary to develop a better characterisation of antibiotic resistance.

## Ethical Approval

This study without involving any patient or human, that no need to have ethical clearance.

## Consent

This study without involving any patient or human, that no need to have inform consent

## Registration of Research Studies

This study only case report from vegetable that not involving human, that do not comply the Helsinki Declaration.

## Guarantor

The Guarantor is the one or more people who accept full responsibility for the work and/or the conduct of the study, had access to the data, and controlled the decision to publish.

## Author contribution

SAM conduct the research and draft the manuscript; W, TA, MR were supervised the research, and finalized the manuscript.

## Funding

This study was funded by Ministry of higher education of libya.

## Provenance and peer review

Not commissioned, externally peer reviewed.

## Declaration of competing interest

All author declare have no conflict of interest
